# Rapidly Exploring Random Tree Algorithm-Based Path Planning for Worm-Like Robot

**DOI:** 10.3390/biomimetics5020026

**Published:** 2020-06-05

**Authors:** Yifan Wang, Prathamesh Pandit, Akhil Kandhari, Zehao Liu, Kathryn A. Daltorio

**Affiliations:** Department of Mechanical and Aerospace Engineering, Case Western Reserve University, Cleveland, OH 44106, USA; yxw780@case.edu (Y.W.); pxp309@case.edu (P.P.); axk751@case.edu (A.K.); zzl@case.edu (Z.L.)

**Keywords:** soft robotics, worm-like robot, path planning, RRT

## Abstract

Inspired by earthworms, worm-like robots use peristaltic waves to locomote. While there has been research on generating and optimizing the peristalsis wave, path planning for such worm-like robots has not been well explored. In this paper, we evaluate rapidly exploring random tree (RRT) algorithms for path planning in worm-like robots. The kinematics of peristaltic locomotion constrain the potential for turning in a non-holonomic way if slip is avoided. Here we show that adding an elliptical path generating algorithm, especially a two-step enhanced algorithm that searches path both forward and backward simultaneously, can make planning such waves feasible and efficient by reducing required iterations by up around 2 orders of magnitude. With this path planner, it is possible to calculate the number of waves to get to arbitrary combinations of position and orientation in a space. This reveals boundaries in configuration space that can be used to determine whether to continue forward or back-up before maneuvering, as in the worm-like equivalent of parallel parking. The high number of waves required to shift the body laterally by even a single body width suggests that strategies for lateral motion, planning around obstacles and responsive behaviors will be important for future worm-like robots.

## 1. Introduction

Due to soft characteristics, nonholonomic constraints, limits on reachable space and the high number of degrees of freedom (DOF), navigating and path planning for worm-like robots can be difficult [1,2,3]. Inspired by earthworms, worm-like robots locomote by changing the body shape of each segment. The segment shape is constrained such that extension in length is coupled with contraction in diameter, and contraction in length is coupled with expansion in diameter. By actuating the segments in a given sequence, the robot can generate a spatial peristalsis wave to move either forward or backward [4].

**Figure 1:**
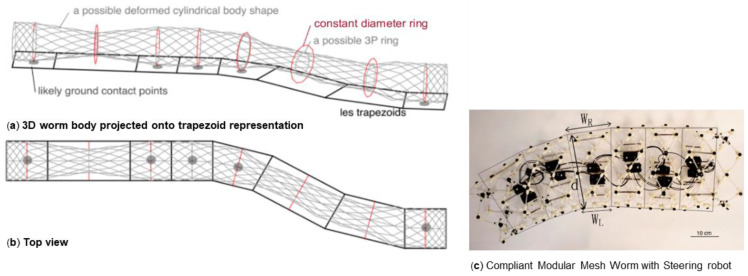
A typical worm-like robot can be simplified as a series of 2D trapezoids [3]: (**a**) Simulated 3D structure of a worm-like robot (gray mesh) and its 2D trapezoid representation (black lines). (**b**) The structure and trapezoids projection viewed from the top. (**c**) 2D trapezoids (black lines) shown overlaid on a picture of Compliant Modular Mesh Worm with Steering robot (CMMWorm-S), as viewed from the top.

**Figure 2:**
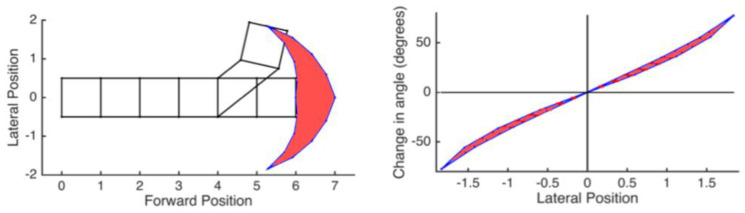
The kinematic constraints we see in our robots limit the amount and type of possible turning [3]. If each wave can involve only two segments due to power density constraints, the front segment can only reach a limited space of points after each wave (**left**, reachable area is outlined in blue with red filling as examples) and at point, the resulting orientation is narrowly constrained (**right**).

To turn, the wave must be adjusted so the amplitude is different on the left and right of the segment. We have previously shown that even if the robot’s structure is simplified as a series of 2D trapezoids (Figure 1), changing from straight-line locomotion into a turn requires multiple, unique waves that are not periodic. This is in part because the shape of the segments can only be changed within certain bounds as shown in Figure 2 because of the limit of the segment deformation. As a result, both the length traveled and the angle turned for each wave are limited. Turning angle also limits traveling distance per wave: The more the robot turns in a certain wave, the less distance it can move [3]. Our previous design, the compliant modular mesh worm robot with steering (CMMWorm-S) is typical of such robots [4].

Through this study, we are intending to develop and implement an efficient and robust path planning method for the CMMWorm-S robot as well as other robots following similar locomotion patterns [5]. To achieve such a goal, a modified rapidly exploring random tree (RRT) algorithm has been developed to form a feasible path from an initial position and orientation to a desired position and orientation. As with original RRT, motion is planned according to the decision trees which are expanded into new sampled states [6]. However, in our modified method, we are going to determine the equation of a curve based on the initial and desired configuration of the robot and make the robot follow the curve.

### Related Work

Although path planning for soft robots is a relatively new topic, there have been several approaches to solving similar problems.

A considerable amount of well-developed searching algorithms, most notably A-star and RRT, have been applied to path planning of autonomous and semi-autonomous vehicles and Unmanned aerial vehicles (UAVs) [7]. Correspondingly, methods to smooth the path between waypoints of the path planning result have also been introduced depending on the specific kinetic constraints and requirements of the given moving subject [8]. Clothoid and spline curves are often applied when the smoothness of acceleration is required. Simpler curves such as polynomial and Dubins curves are more preferred when the resources for runtime calculation are limited [8,9]. In contrast to autonomous vehicles, worm-like robots have less velocity and each segment has a stationary support or anchoring phase while other segments locomote. As a result of these characteristics, the smoothness of acceleration has little impact on kinematics. A simpler and less costly path smoothing method is therefore preferred. As a result, the ellipse is selected for our case which has less maximum curvature than Dubins method.

Here we assess whether elliptical curves are appropriate approximations for the specific nonholonomic constraints that arise from peristaltic locomotion.

For snake-like robots, techniques based on serpenoid curves and genetic algorithms have been proposed in which the range of path and curve deviation are used as constraints to compute a path [10]. Planning a path for snake-like robots has also been solved with potential energy methods [11].

Algorithms have also been presented for deformable robots. Gayle et al. [12] presented an algorithm for path planning of deformable robots by using the probabilistic roadmap method. In this algorithm, they used constraints like preserving volume in order to make corrections and make an appropriate path. In [13], roadmaps are built for deformable volumes. The nodes of these roadmaps are equilibrium configurations of volume under constraints and hence find the path by searching the roadmap. In [14], the probabilistic roadmap planner finds a path based on the Bezier surface and energy function. In [15], the path is formed in two stages: first, the approximate path is formed without considering collisions and second, the path is corrected by deforming the robot wherever there are collisions. In soft robotics, there is also research focused on an opposite principle: finding a path where the robot contacting the edge of the obstacles is considered having the lowest cost. In such a case, the soft robot can utilize such contacting points to enhance its locomotion [16,17].

Meanwhile, research has been done on the locomotion of worm-like robots and its relation to the robot properties such as size, stiffness and deforming pattern [18,19]. Our simulation models shown in the following section follows the slip elimination criteria during locomotion. This has been previously published [19] and states that in order for a peristaltic device to turn without slipping, the actuation pattern must change each wave depending on the previous configuration of the robot. Such waveforms have been termed as non-periodic waveforms.

To our knowledge, though different approaches on path-planning for the soft robot have been developed, no other research has focused on a similar topic as of this paper: path-planning solution to deal with the complicity and nonholonomic constraints of worm-like robot.

In order to find an appropriate pathfinding method for the worm-like robot, we started from two kinds of simple algorithms (RRT (Algorithm 1) and elliptical path generation (Algorithm 2)). Then we combined those two algorithms (combined RRT ellipse (Algorithm 3) to benefit both of their advantages. Based on such combination, we introduced a more advanced algorithm (enhanced combined RRT ellipse (Algorithm 4)) with some helpful improvements. A brief overview of these 4 algorithms is shown in Table 1.

The rest of this paper is organized as follows: Section 2 will provide more details about the applied robot path planning algorithm; Section 3 will show the experimental results of the presented algorithm in a simulated environment; Section 4 will introduce a discussion of the different algorithms and their potentials; Finally, Section 5 will provide the summarized conclusions.

## 2. Methods: Pathfinding Algorithms

### 2.1. Random Trees (RRT)

In this classical RRT algorithm, we initialize the configuration of the robot and the maximum number of iterations. The configuration includes the coordinate *P*(*x,y*) and heading angle of its first segment’s center of mass; length of the left and right segments of trapezoids (*W_L_* and *W_R_*). At each iteration, a random coordinate and then a configuration of the robot closest to that coordinate is selected and added to the decision tree. The configuration of the robot is selected based on its constraints as shown in Figure 2. When finding such configurations, previous configurations from the decision tree are taken into consideration. Those steps will repeat until (1) the tree has reached the goal; (2) the number of iterations has reached its preset maximum value. In case (2), it selects the point and configuration that is closest to the goal.
**Algorithm 1** RRT  Input: Initial and desired configuration of the robot, the maximum number of samples, *N_max_*  Output: Tree, *T*  1.Add initial configuration as a node *C_1_* to the tree, *T*  2.For *i* = 1 to *N_max_*:
*R* = Random Coordinate (*x, y*)Pick existing configuration *C_p_* of the tree whose Coordinate *P* is closest to *R* in the geometric distance.Calculate (*W_L_, W*_R_) that locomotes the robot from *P* to *R* where the geometric distance to *R* reduces the most to generate a new set of configurations {*C*}.Add {*C*} to treeStop if any configuration of {*C*} is within a tolerable error range for both geometric distance and head orientation.  3.Return *T*

### 2.2. Elliptical Path Generation

RRT can be time-consuming as it explores many configurations of the robot. In order to reduce the time cost, we intend to find a simple mathematical expression of the path that can smoothly connect the initial and final configuration of the robot.

In this approach, instead of randomly exploring spaces to form a path for a robot, we are going to determine an equation of curve for the robot to follow. The equation of a curve is determined on the basis of the initial and desired configurations of the robot. The constraints are as follows:

- The robot is tangent to the curve at the start coordinate
- The robot is tangent to the curve at the goal coordinate
- The start coordinate of the head center of the robot satisfies the equation of the curve.
- The goal coordinate of the head center of the robot satisfies the equation of the curve.

We choose an elliptical curve as the path for the robot to follow. The general equation of the ellipse (in terms of coordinate (*x,y*)) can be written as:

(1) $$\frac{{\left(x-h\right)}^{2}}{a^{2}}+\frac{{\left(y-k\right)}^{2}}{b^{2}}=1$$

(2) $$\{\begin{array}{c} x=h+a\cdot cos(t)\\ y=k+b\cdot sin(t) \end{array},t\in \left(0,2\pi \right]$$

Equations (1) and (2) are equivalent. There are four unknown parameters in the ellipse’s equation which are the center of the ellipse (*h, k*), major axis *a* and minor axis *b*. These unknowns can be determined by the above four constraints. As mentioned in Figure 2, our robot has a range of angles it can turn in a single wave. Once we have the equation of the elliptical curve, we determine the angle by which the robot should turn per peristaltic wave. These angles are chosen such that the robot follows the generated elliptical path with minimal deviation, thereby minimizing errors. Once the robot reaches the goal, it stops following the ellipse.
**Algorithm 2** Elliptical path generation  Input: Initial and desired configuration of the robot  Output: List of angles, *W*  1.Apply Equation (1) to determine *a,b,h,k* based on initial and final configuration such that:$\{\begin{array}{l} \frac{{\left(x_{r}-h\right)}^{2}}{a^{2}}+\frac{{\left(y_{r}-k\right)}^{2}}{b^{2}}=1\\ \frac{\left(x_{r}-h\right)}{a^{2}}+\frac{\left(y_{r}-k\right)\cdot m_{r}}{b^{2}}=0 \end{array},\;\;\{\begin{array}{l} \frac{{\left(x_{d}-h\right)}^{2}}{a^{2}}+\frac{{\left(y_{d}-k\right)}^{2}}{b^{2}}=1\\ \frac{\left(x_{d}-h\right)}{a^{2}}+\frac{\left(y_{d}-k\right)\cdot m_{d}}{b^{2}}=0 \end{array}$where *(x_r_, y_r_)* is the robot’s center coordinate of the head of initial configuration, *m_r_* is the robot’s tangent of the orientation of initial configuration and *(x_d_, y_d_)* is the center coordinate of the head of desired goal configuration, *m_d_* is the tangent of the orientation of the desired configuration  2.Current configuration, *C_i_* = Initial configuration, *I*  3.While *C_i_* ≠ Desired Configuration, *D*:
Execute set of angles to follow ellipse to generate a new set of configurations {*C*}Select new configuration, *N* from {*C*} which closely satisfies the ellipseAdd angle of *N* to list of angles, *W**C_i_* = *N*  4.Return *W*

### 2.3. Combined RRT Ellipse

In some special cases, a single elliptical path from initial and final configuration may not exist. For example, from the initial condition shown in Figure 4, if the goal is in the first quadrant and the desired orientation is zero degrees. In this case, an ellipse cannot be formed due to geometrical constraints. However, it is possible to reach the goal with multiple ellipses. Hence an algorithm is needed which not only forms multiple ellipses but also follows them. Thus, combining these two approaches wherein we take the robot’s constraints into consideration and check whether the robot is deviating from generated ellipse and while checking whether the robot is getting stalled.

In this method, we randomly select major axis *a*, minor axis *b* and direction of the ellipse, and direction of the worm. After determining the ellipse from a, b and direction of the ellipse (finding center of ellipse (*h, k*)) we let the robot follow the ellipse until it has completed the ellipse, or drifted from the ellipse, or is stalled. In that case, we select a new configuration by which it can reach the goal with minimum distance and add that configuration to the decision tree. Finally, after all the iterations we select the configuration which is closest to the goal and execute the path.

In RRT, in order to add a new configuration to the tree, we selected the configuration that is close to the goal. In this method, we are estimating the remaining distance after each wave and adding it to the distance traveled so far by the robot. In order to estimate the remaining distance, we are determining a new ellipse after each wave based on constraints mentioned in the elliptical path generation. Once we find parameters of the ellipse, we estimate its arc length from its current position to the goal. The arc length is determined as:

(3) $$L_{EA}=b\cdot \int_{0}^{\theta_{d}}\sqrt{1-\varepsilon \cdot {sin}^{2}\left(\theta_{d}\right)}{\;d\theta }_{d}$$

where $L_{EA}$ is the Elliptical Arc length, $\theta_{d}$ is the angle between the radii from position to goal and $\varepsilon =1-\frac{a^{2}}{b^{2}}$

(4) $$L_{total}=L_{traveled}+L_{EA}$$

where $L_{total}$ is the estimated total distance, $L_{traveled}$ is the recorded traveled distance and $L_{EA}$ is the Elliptical Arc length

As previously mentioned, for some special cases an ellipse path is not feasible. In such cases, we eliminate the constraint that the robot is tangent to the start coordinate. From the remaining constraints, we determine a circle and hence compute the circular arc length. In this case, in order to determine estimated arc length, we multiply the circular arc length with a penalty and then add it to the distance traveled. By doing this, it is less likely to select that configuration by which an elliptical path is not possible.

(5) $$L_{total}=L_{traveled}+\omega \cdot L_{arc}$$

where $L_{total}$ is the estimated total distance, $L_{traveled}$ is the recorded traveled distance, ω is a manually selected penalty weight and $L_{EA}$ is the circular arc length.
**Algorithm 3** Combined RRT and elliptical path  Input: Initial and desired configuration of the robot, the maximum number of samples, *N_max_*  Output: Tree, *T*  Add initial configuration to the tree, *T*  1.For *i*=1 to *N_max_*:    a.Randomly choose *a*,*b*, direction of ellipse (clockwise/anti-clockwise), direction of ellipse    b.Sample a configuration, *C_s_* from the tree, *T*    c.Apply Equation (2) to determine center coordinate of the ellipse (*h,k*) based on:   $\{\begin{array}{l} \tan\left(\theta \right)=\frac{-b}{{a\times \theta }_{s}}\\ x_{n}=h+a\cdot cos\left(\theta \right)\\ y_{n}=k+b\cdot sin\left(\theta \right) \end{array}$     (*x_n_,y_n_*) is the coordinate of the head of *C_s_* and *θ_s_* is the tangent of the orientation of *C_s_*    d.Execute a set of points from *C_s_* which the robot can follow and closely satisfy the ellipse to generate a new set of configurations {*C*}    e.Find best of {*C*} based on Equations (4) or (5) and add it to *T*  2.Return *T*

### 2.4. Enhanced Combined RRT Ellipse

**Figure 3:**
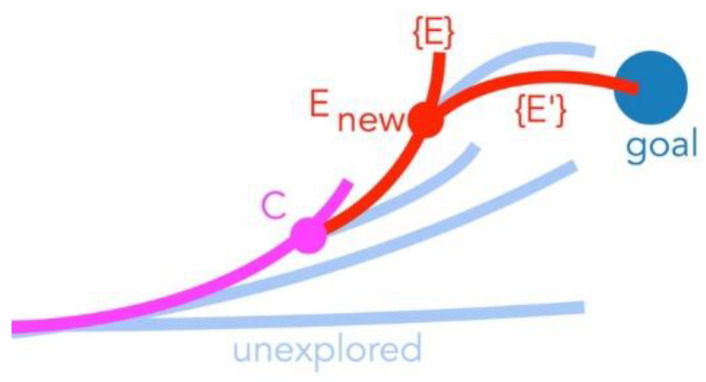
Checking the second ellipse, as in Algorithm 4 is important because it enables more direct search for desired configuration (blue circle) within a single step (red lines) before exploring new branch additions (light blue) to existing tree (magenta).

In this method, the procedure is the same as the combined RRT and elliptical path generation until it selects the configuration to add it to the tree. Once the remaining path from the current configuration to the final goal can be generated as an ellipse, it directly completes the path without introducing errors instead of randomly selecting waypoints. After several iterations, we get many solutions. Among those solutions, it selects the path by which it requires a smaller number of waves (Figure 3).
**Algorithm 4** Enhanced combined RRT and elliptical path  Input: Initial and desired configuration of the robot, the maximum number of samples, *N_max_*  Output: Tree, *T*  1.Add initial configuration to the tree, *T*  2.For *i* = 1 to *N_max_*:
Repeat Algorithm 3 from 2.a to 2.d to get {*C*} from configuration *C_s_*Find best of {*C*}, *C_new_*, that gives least estimated distance based on Equation (4) or (5)If *C_new_* used Equation (5):
Repeat Algorithm 2 from 1.a to 1.d with initial condition assigned as *C_new_* to create a new ellipseExecute a set of points from *C_new_* which the robot can follow and closely satisfy the ellipse to generate a new set of configurations {*C’*}Find best of {*C’*} based on Equations (4) and add it to *T*If the robot reaches the goal position, then immediately return *T*  Else:  Add *C_new_* to *T*    3.Return T

In other words, the difference between Algorithm 3 and 4 is that Algorithm 4 enables two new ellipses to be added in a single step if the goal is reached from the best of first ellipse.

## 3. Experimental Results

All algorithms in Table 1 have been tested in MATLAB simulation environment with the constraints described in Section 1. In the examples shown, the initial condition of the robot is horizontal for all segments. The error between the robot and the goal is evaluated based on the equation:

(6) $$E=\sqrt{{{(x}_{d}-x_{r})}^{2}+{(y}_{d}-y_{r}{)}^{2}}+K\cdot |\alpha_{d}-\alpha_{r}|$$

where *E* is the error between the robot and the goal (in centimeter); *(x_r_, y_r_)* and $\alpha_{r}$ are the robot’s center coordinate and heading angle in radian of the head of the robot’s current configuration; *(x_d_, y_d_)* and $\alpha_{d}$ are the center coordinate and heading angle in radian of the head of desired goal configuration; *K* is a manually chosen conversion weight indicating how critical the angle accuracy is to a specific path-planning problem (with a unit of centimeter per radian). The parameter *K* can be seen as a tradeoff between distance error and angular error is meant to be manually selected depending on scenario requirements; the more critical angular error matters to a specific case, the higher value *K* should be. For the examples in this paper, the *K* is always assigned to 1. The robot is considered reaching the goal once *E* is smaller than 2.

**Figure 4:**
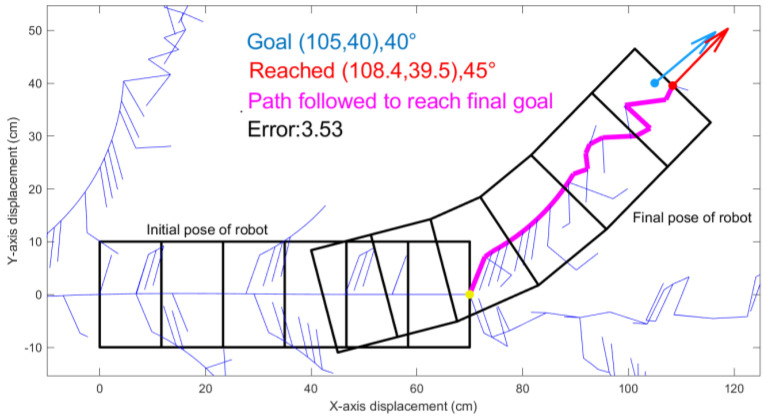
An RRT generates a random tree (thin blue lines) from the initial position (yellow dot) toward the desired goal position and orientation (blue). After 1000 iterations, it reaches the best position shown in red.

Specifically, as shown in Figure 4, the initial configuration of the robot is such that its rear edge is on the *y*-axis and its centerline is on the *x*-axis. The initial and goal coordinates are based on the coordinate of the center of the head of the robot.

### 3.1. Rapidly Exploring Random Trees

As expected, RRT creates an expanding tree that considers discrete movements of individual waves. However, these waves appear inefficient. For example, we considered a goal just 2 body widths away from centerline here at position (105,40), with angle change of 40° orientation as blue dot and arrow in Figure 4. The yellow dot represents the initial center coordinate of the robot. The blue lines show the decision tree *T*, that traces the coordinates of the center of the first segment or head of the robot for 1000 iterations. The best resulting path is shown in magenta in Figure 4.

### 3.2. Elliptical Path Generation

**Figure 5:**
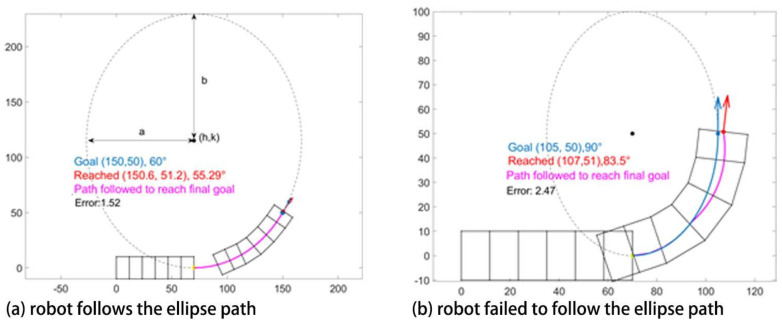
Examples of elliptical path generation, blue dot and arrow are the desired position and orientation, red dot and arrow are the reached position of the simulated robot. The blue curve is the ellipse generated and pink is the path executed. In case (**a**) where robot follows the ellipse, blue and red arrow almost coincide which shows that the robot has successfully reached its goal. In case (**b**) the robot is not able to follow the ellipse, and thus the pink and blue curves do not coincide because the elliptical axis length in the lateral direction is too small.

Following an ellipse is a faster way to calculate how to get from initial to the desired configuration, but an ellipse is only an approximation of the path the robot can follow. Here, starting from the same initial conditions as above, the ellipse can be accurately followed along some curves. (such as Figure 5a.) However, as seen in Figure 5b, the robot may fail to follow the ellipse in certain scenarios when the ellipse is too small. This is due to the fact that the robot’s kinematic constraints are a function of the shape of the entire body of the robot, so they are not taken into account when determining ellipse parameters. In Figure 5, the blue curve is the generated ellipse based on the initial and final configurations of the robot. The pink curve is the path robot has followed.

### 3.3. Enhanced Combined RRT Ellipse

**Figure 6:**
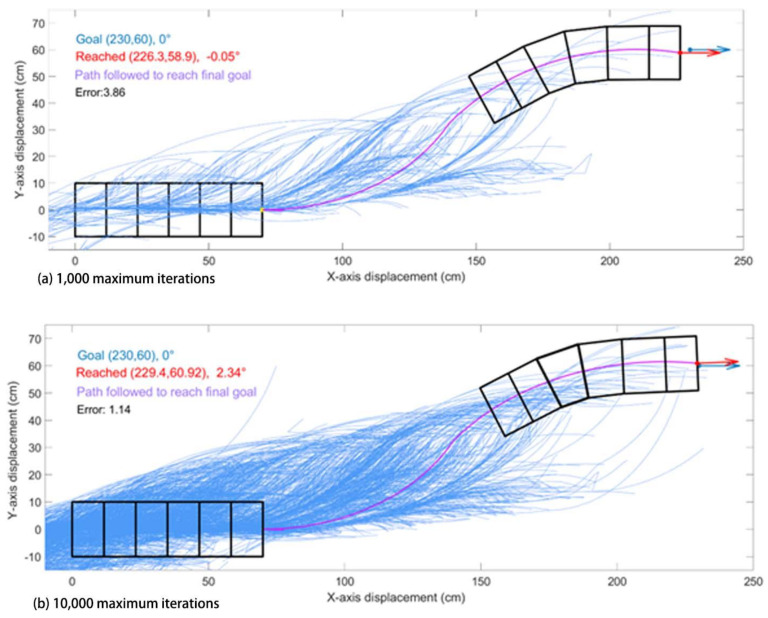
Examples of combined RRT ellipse where the tree of random elliptical paths (blue curves) are generated with (**a**) 1000 maximum iterations; (**b**) 10,000 maximum iterations. The pink curve is the path the robot has chosen from the tree. Blue dot and arrow are the goal position and orientation. Red dot and arrow are the reached position and orientation of the robot. In this case, the robot successfully approached the goal with 10,000 maximum iterations.

Using this algorithm, the robot approaches the goal in most cases. However, the RRT method is computationally expensive as compared to the Elliptical Path Generation methods. In order to reach the goal with a smaller error, many more iterations are required. This is shown in Figure 6 where with maximum iterations set to 1000 it approaches close to the goal, but to get even closer 10× more iterations are required.

### 3.4. Enhanced Combined RRT Ellipse (ECRE)

**Figure 7:**
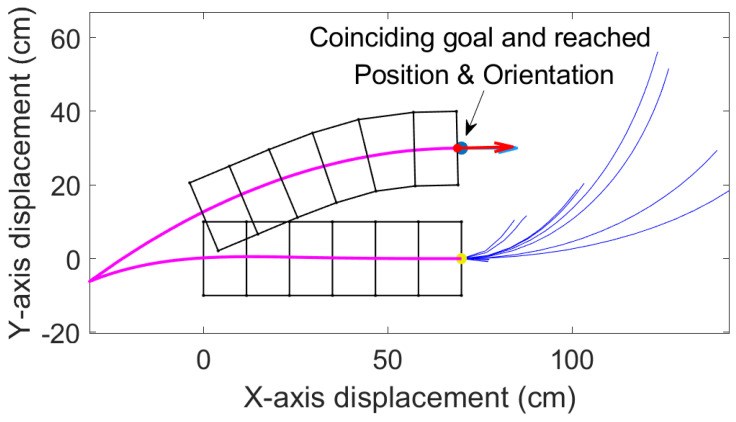
Here the enhanced combined RRT ellipse is used to reposition the first segment at a lateral offset in a parallel orientation, where the blue curves are the tree of randomly generated elliptical paths and the pink curve is the path chosen from the tree. Blue dot and arrow are goal position and orientation. Red dot and arrow are the reached goal and orientation. Blue and red arrows coincide which shows that the robot accurately reached the preset goal.

This algorithm is much faster compared to the above three in yielding results. We demonstrate this in a challenging situation for worm-like robots in which the robot needs to reposition the body parallel to the original position but offset laterally by 1.5 body widths. In other words, this is a worm-like robot equivalent to a “parallel-parking” problem. As shown in Figure 7, the best solution involves moving backward first and then moving forward again. With enhanced combined RRT ellipse, we can find this solution in 15 iterations and the goal and reached position almost coincide. Forward moves are also considered (as shown in blue lines).

In positions where multiple solutions are found after the set number of iterations, the path that requires the fewest number of waves is chosen.

### 3.5. Algorithm Efficiency Comparison

**Figure 8:**
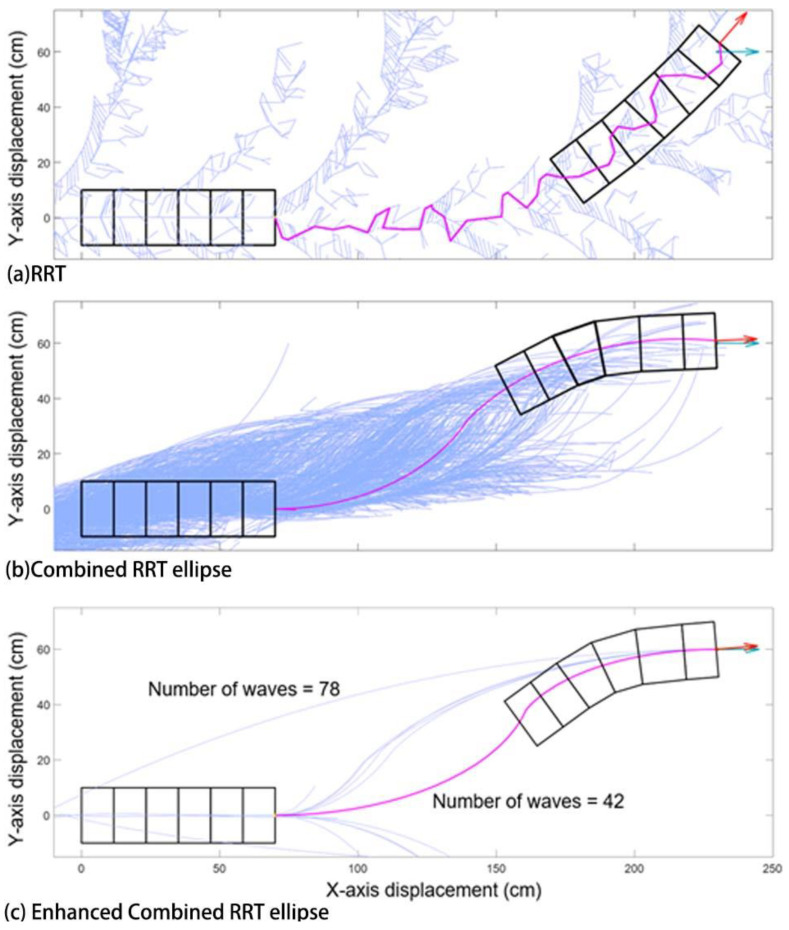
Results are compared when (**a**) RRT, (**b**) Combined RRT ellipse and (**c**) Enhanced combined RRT ellipse is used to reach the same goal which is (230, 60) and orientation is 0 degrees (horizontal). Blue lines are the tree and pink is the path chosen from the tree. (**a**) RRT where the blue and red arrow do not coincide after 10,000 iterations, indicating that the robot was unable to reach its goal position and orientation. (**b**) Combined RRT Ellipse where it has reached the goal within 10,000 iterations (**c**) Enhanced combined RRT ellipse where it has successfully reached the goal with multiple solutions (paths). The algorithm then picks the best path based on the least number of waves to the desired goal position.

As shown in Figure 8, both classic RRT and combined RRT Ellipse require a massive number of iterations to eventually approach the goal. For RRT, as seen in Figure 8a, even after 10,000 iterations, the robot has not reached the goal position and orientation, so the robot has chosen the configuration which is closest. The tree of combined RRT Ellipse is much closer to the goal compared to the one in classic RRT within the same maximum iterations. This is because, in RRT, just one wave is explored at each iteration; whereas, in combined RRT Ellipse, multiple waves are calculated in each iteration.

Our research evaluates the total wave needed to follow the path as an indicator of efficiency. This value is proportional to total locomotion time since the wave of earthworm robot (e.g., our CMMWorm-S robot) is normally generated in a certain temporal pattern. In a case where the energy consumption is more critical, some other indicators like the cost of transport can be introduced to replace the total waves in the cost function during pathfinding.

The advantage of enhanced combined RRT ellipse is that the result is accurate as of the combined RRT Ellipse method, but requires much fewer iterations to find solutions, such that in fact multiple solutions are found and that the planner can pick the best one, shown in Figure 8c. The one with fewest waves is selected. As a result, it takes less total time to find a solution, even considering the fact that each iteration takes slightly longer (Table 2).

**Figure 9:**
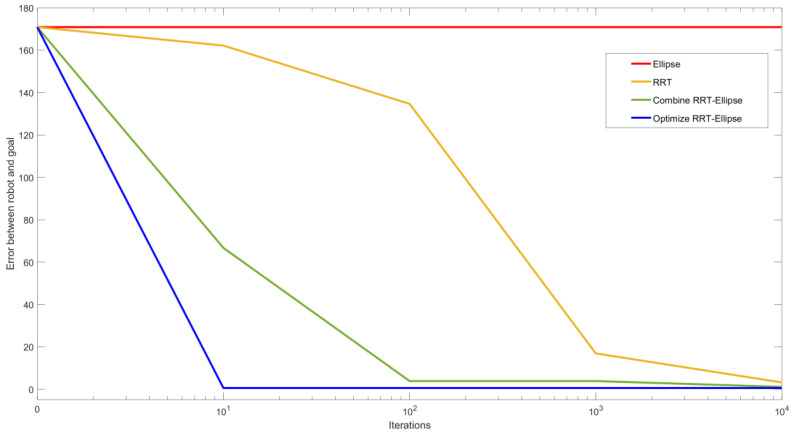
The error between reached solution and goal decreases with increasing iterations for all four algorithms where the goal is the same as in Figure 8. Except for the single Ellipse approach, all the algorithms converge, however, enhanced combined RRT ellipse converges faster (reaching goal within 10 iterations) whereas combined RRT Ellipse requires 10,000 iterations to achieve a closer solution accuracy.

Figure 9 shows how efficient the enhanced combined RRT ellipse algorithm is. It can be seen that as we increase the number of iterations, the tree is closer to the goal (except the ellipse algorithm. The ellipse algorithm alone will not work for this goal as a single elliptical curve is not possible given those constraints). The enhanced combined RRT ellipse showed its extraordinary advantage on path planning amongst these 4 compared algorithms as distance approximately drops to zero within 10 iterations whereas RRT is not able to reach the goal even after 10.000 iterations.

Table 2 depicts the comparison of time elapsed in each algorithm with more details. It can be seen that RRT consumes the least time to conduct one iteration, but as it requires massive iterations the total time consumption is far higher than other methods. Overall, the enhanced combined RRT ellipse cost significantly less time to stably find the path. The 10 s of total time consumption still holds the potential to be further reduced by a deeper code optimization (disabling path-image display, porting to higher execution efficient coding language). As for the low-velocity robots, such timescale is acceptable for runtime planning in stationary environments. For example, the 6-segment CMMWorm-S robot typically spends 18 s to finish a whole-body wave under 3 × 1 wave pattern. In such case the computation time is about 1% of the total time to follow a path that requires 42 waves to reach the goal [3,19].

### 3.6. Path Analysis of Reachable Space

**Figure 10:**
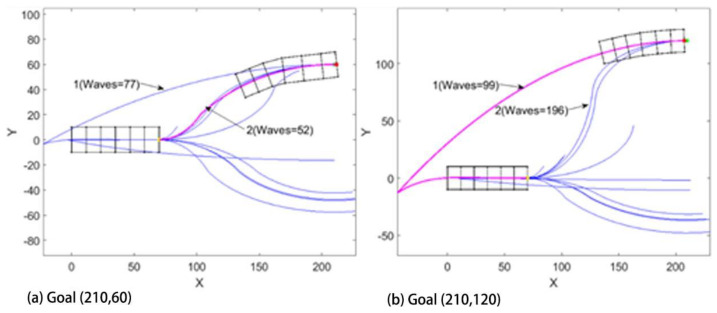
The Enhanced combined RRT Ellipse method searches both forward and backward and returns the path that costs least waves: (**a**) Goal (210,60). The path taken is initially turning left in the forward direction and then turning right. (**b**) Goal (210,120). The path taken is turning right in the backward direction and then turning right in the forward direction.

**Figure 11:**
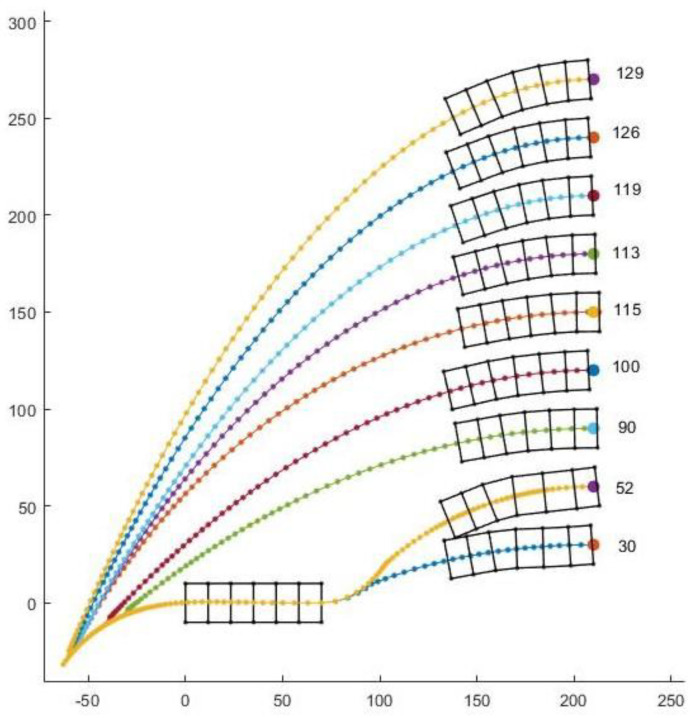
For a set of goal positions with increasing lateral offset, different paths are taken to reach the goal where each dot represents the position of the center head after each wave. The numbers in the figure show the number of waves required to reach the corresponding goal. A qualitative difference is clear after the first two paths in which it becomes better for robot to reverse direction before moving forward in the x direction.

It may seem that paths might scale with elliptical parameters, allowing previous solutions to similar problems to predict new results, however, there is a bifurcation in path strategy that corresponds to cases when the worm robot could reach its goal in fewer waves by moving backward first. In other words, there is a qualitative difference in the path strategy as the goal state varies laterally. In Figure 10a,b, the goal is of similar type that is in the third quadrant and the desired angle is zero, but the coordinate is different in both cases. In Figure 10a, we can see that path 1 is first turning right backward and then turning right forward and path 2 is forward left and forward right. Out of these two paths, the second path has the least number of waves, so it executed path 2. Now in Figure 10b, we get similar traits of paths but in this case, it has chosen the first path as the solution. That’s because in the figure while using paths 2 and 3, it has to take a tighter turn while changing the direction, hence the change in angle after each wave during the change in direction is very small thus increasing the number of waves and taking more time. Figure 11 shows different paths the robot takes where the x-coordinate is constant, and we have kept changing the y-coordinate.

**Figure 12:**
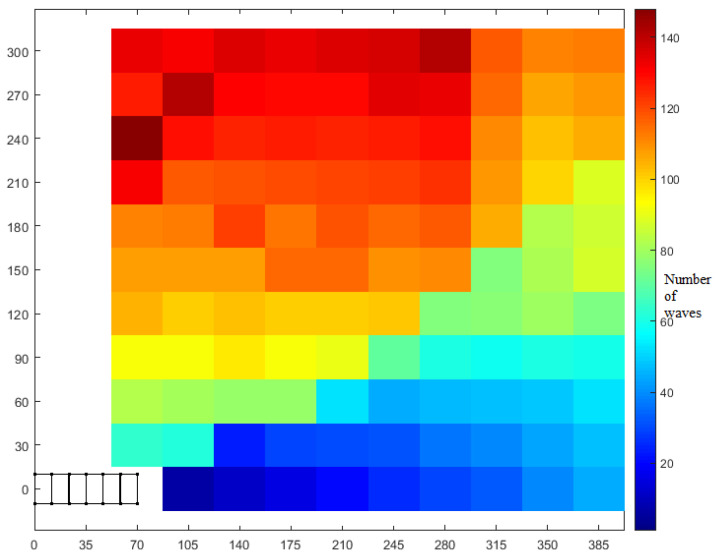
Enhanced combined RRT ellipse enables computation of the number of waves required to arrive at points in the space around the robot with goal orientation horizontal right (same as original). Each color in the cell denotes the number of waves required to reach that position. As expected, points farther from initial condition in each direction require more waves. A diagonal pattern of color discontinuities demonstrates the boundary between points that are best reached with forward motion (lower right) and points that are best reached by reversing first (upper left).

Thus, with enhanced combined RRT ellipse, we can characterize each point in the nearby reachable space around the robot in terms of the number of waves required. In Figure 12, we demonstrate the total required waves to reach each goal position with the 0° goal front segment angle (horizontal rightwards). This seems most likely to be relevant for the case of a worm robot approaching an entry point such as a hole in the wall that must be entered orthogonally.

In Figure 12, 110 coordinates (center of each colored block) are alternately assigned as goal position with the horizontal right as orientation. The initial position of the head center is (70,0). The x-coordinates are with an interval of 35 from the initial position and y-coordinates are with an interval of 30. It can be seen that the robot takes the minimum number of waves to keep on increasing as we go from the bottom right corner to the top left corner. In this plot, we iterated for 10 iterations and stopped if path was found, but continued until a sufficient result was found as needed (for upper right corner where the number of waves is higher).

This figure shows how accurately this kind of worm robot would need to observe an entry point without having to back up. When the goal is 5 body lengths away, if the entry point is not within 2.5 body lengths (or 9 body widths), the robot will need to reverse to find the goal. Only the first quadrant data is plotted, but this is symmetric for left and right turns, or for vertical turns, defining a conical boundary that can be observed by the sharp increase in the number of waves as *y*-axis increases.

## 4. Discussion

We have found a modification of RRT that enables the computation of appropriate plans for a worm-like robot to travel to goals in nearby space without slipping. This is an improvement over RRT alone, which takes a long time to find solutions that are not smooth. The basis of this approach is in following elliptical arcs. These are not sufficient alone because (a) the robot does not always follow the ellipse and (b) in some cases, there is no possible elliptical equation based on the initial and desired configuration. By adding the ellipse to RRT, the tree is closer to the goal than in RRT alone. We see that after 10,000 iterations, the tree reaches the goal. Since the shape of the trapezoids must be within the bounds shown in Figure 2, the range at which the worm-like robot can turn is limited. This is the main reason why the tree requires a large number of iterations to reach the goal. For the specific problem of planning for worm-like robots, we have enhanced the process by following two ellipses in a single iteration, thereby computing the path in fewer iterations, which make it possible to commit path planning in real-time and redo such path-planning after each wave to adjust module inaccuracy and environmental influence.

The limitations on turning are not unique to our worm robots but could be common among soft robots where turning depends on body deformation. While in many cases the planning may be reactive or may take advantage of environmental features (such as an earthworm robot following the curvature of a tube), it seems desirable that such robots also be able to cross open terrain precisely in order to perhaps enter the next confined space opening. Understanding the planning constraints not only will help such robots efficiently align their bodies for subsequent stages but also can guide and benefit worm-like robot design to improve its maneuverability. Alternatively, this work may also show the value of omnidirectional movement mechanisms even in robots with long narrow form factors. We expect these considerations to be especially valuable in even more cluttered planning problems with obstacles, which will be addressed in future work.

## 5. Conclusions

Our study aimed to build and apply the challenging path planning for robots that are using soft body locomotion, like the worm robots in (Figure 1). Each segment has a limited range of motion (Figure 2). This results in nonholonomic constraints, like for a rolling wheel, for each anchoring segment. Furthermore, the turns possible depend on not only the configuration of a single segment but of all the segments on the ground. This makes the robot in a way “hyper-nonholonomic”. Our proposed solution is to generate a reliable smooth path (Table 1 and Figure 3). The result is a connected tree of reachable configurations (Figure 4). If the arcs are too tight, the worm robot will only be able to follow part of the arc (Figure 5). As expected, running the algorithm for additional iterations makes the final configuration closer to the goal (Figure 6). Sometimes reversing is required (Figure 7). Our enhanced combined RRT ellipse method can find multiple smooth paths faster than the original RRT or combined RRT Ellipse (Paths are shown in Figure 8, convergence over iterations shown in Figure 9, path generating time consumption shown in Table 2). When two paths are possible, we choose the path with the least waves (Figure 10). As the goal position is moved laterally, the best path requires reversing direction first (Figure 11). We used our algorithm to determine the minimum number of waves to reach each position in local space from the initial position (Figure 12), the diagonal discontinuity between colors indicates the boundary where reversals are required.

## Figures and Tables

**Table 1:** Implemented algorithms.

Algorithm	Guaranteed Goal Convergence	Smooth Path	Total Computational Time
RRT (random tree of individual waves growing toward the goal)	√		high
Ellipse (single ellipse path tangential to start point and goal)		√	N/A
Combined RRT ellipse (random tree of ellipses growing toward goal)	√	√	high
Enhanced combined RRT ellipse (random tree of ellipses and when waypoints are close to goal, ellipse endpoints are set at goal)	√	√	low

**Table 2:** Comparison of time elapsed for algorithms where the goal is the same as in Figure 8.

Algorithm	Maximum Iterations Tried	Reach the Goal?	Final Error	Time for One Iteration (s)	Total Time Elapsed (s)
RRT	10,000	No	3.1984	0.76	3937
Ellipse	1	No	170.9	3.23	3.23
Combined RRT Ellipse	10,000	Yes	1.1109	3.69	47719
Enhanced Combined RRT Ellipse	10	Yes	0.5697	4.85	312

## References

[1] Trivedi D., Rahn C.D., Kier W.M., Walker I.D. (2008). Soft Robotics: Biological Inspiration, State of the Art, and Future Research. Appl. Bionics Biomech..

[2] Polygerinos P., Correll N., Morin S.A., Mosadegh B., Onal C.D., Petersen K., Cianchetti M., Tolley M.T., Shepherd R.F. (2017). Soft Robotics: Review of Fluid-Driven Intrinsically Soft Devices; Manufacturing, Sensing, Control, and Applications in Human-Robot Interaction. Adv. Eng. Mater..

[3] Kandhari A., Wang Y., Daltorio K., Chiel H.J. (2019). Turning in Worm-Like Robots: The Geometry of Slip Elimination Suggests Nonperiodic Waves. Soft Robot..

[4] Horchler A.D., Kandhari A., Daltorio K.A., Moses K.C., Andersen K.B., Bunnelle H., Kershaw J., Tavel W.H., Bachmann R.J., Chiel H.J., Wilson S., Verschure P., Mura A., Prescott T. (2015). Worm-Like Robotic Locomotion with a Compliant Modular Mesh. Biomimetic and Biohybrid Systems. Living Machines 2015.

[5] Zhan X., Fang H., Xu J., Wang K.-W. (2019). Planar locomotion of earthworm-like metameric robots. Int. J. Robot. Res..

[6] Lavalle S.M. (1998). Rapidly-Exploring Random Trees: A New Tool for Path Planning.

[7] González D., Pérez J., Milanés V., Nashashibi F. (2016). A Review of Motion Planning Techniques for Automated Vehicles. IEEE Trans. Intell. Transp. Syst..

[8] Scheuer A., Fraichard T. Continuous-curvature path planning for car-like vehicles. Proceedings of the 1997 IEEE/RSJ International Conference on Intelligent Robot and Systems. Innovative Robotics for Real-World Applications. IROS ‘97.

[9] Yang K., Sukkarieh S. (2010). An Analytical Continuous-Curvature Path-Smoothing Algorithm. IEEE Trans. Robot..

[10] Liu J., Wang Y., Ii B., Ma S. Path planning of a snake-like robot based on serpenoid curve and genetic algorithms. Proceedings of the Fifth World Congress on Intelligent Control and Automation (IEEE Cat. No.04EX788).

[11] Ye C., Hu D., Ma S., Li H. Motion planning of a snake-like robot based on artificial potential method. Proceedings of the 2010 IEEE International Conference on Robotics and Biomimetics.

[12] Gayle R., Lin M.C., Manocha D. Constraint-Based Motion Planning of Deformable Robots. Proceedings of the 2005 IEEE International Conference on Robotics and Automation.

[13] Anshelevich E., Owens S., Lamiraux F., Kavraki L.E. Deformable volumes in path planning applications. Proceedings of the 2000 ICRA. Millennium Conference. IEEE International Conference on Robotics and Automation. Symposia Proceedings (Cat. No.00CH37065).

[14] Lamiraux F., Kavraki L.E. (2001). Planning Paths for Elastic Objects under Manipulation Constraints. Int. J. Robot. Res..

[15] Bayazit O.B., Lien J., Amato N.M. Probabilistic roadmap motion planning for deformable objects. Proceedings of the 2002 IEEE International Conference on Robotics and Automation (Cat. No.02CH37292).

[16] Greer J.D., Blumenschein L.H., Alterovitz R., Hawkes E.W., Okamura A.M. (2020). Robust navigation of a soft growing robot by exploiting contact with the environment. Int. J. Robot. Res..

[17] Ozkan-Aydin Y., Murray-Cooper M., Aydin E., McCaskey E.N., Naclerio N., Hawkes E.W., Goldman D.I. Nutation Aids Heterogeneous Substrate Exploration in a Robophysical Root. Proceedings of the 2019 2nd IEEE International Conference on Soft Robotics (RoboSoft).

[18] Kandhari A., Daltorio K.A. A kinematic model to constrain slip in soft body peristaltic locomotion. Proceedings of the 2018 IEEE International Conference on Soft Robotics (RoboSoft).

[19] Kandhari A., Huang Y., Daltorio K.A., Chiel H.J., Quinn R.D. (2018). Body stiffness in orthogonal directions oppositely affects worm-like robot turning and straight-line locomotion. Bioinspir. Biomim..

